# Effectiveness and Safety of Transcatheter Atrial Septal Defect Closure in Adults with Systemic Essential Hypertension

**DOI:** 10.3390/jcm11040973

**Published:** 2022-02-13

**Authors:** Iwona Świątkiewicz, Łukasz Bednarczyk, Michał Kasprzak, Ewa Laskowska, Marek Woźnicki

**Affiliations:** 1 Department of Cardiology and Internal Medicine, Nicolaus Copernicus University in Toruń, Collegium Medicum in Bydgoszcz, 85-094 Bydgoszcz, Poland; lucas.bednarczyk@gmail.com (Ł.B.); medkas@o2.pl (M.K.); laskowskaewa1@gmail.com (E.L.); marekwoznicki@wp.pl (M.W.); 2 Division of Cardiovascular Medicine, University of California San Diego, La Jolla, CA 92037, USA

**Keywords:** atrial septal defect, transcatheter closure, right ventricular reverse remodeling, residual shunt, systemic essential hypertension, echocardiography, adult congenital heart disease, comorbidities, complications, outcome

## Abstract

Concomitant systemic essential hypertension (HTN) in adults with a secundum atrial septal defect (ASD) can unfavorably affect the hemodynamics and transcatheter ASD closure (ASDC) effects. This study aims to assess the effectiveness and safety of ASDC in adults with HTN in real-world clinical practice. Right ventricular (RV) reverse remodeling (RVR) and the lack of a left-to-right interatrial residual shunt (NoRS) in echocardiography 24 h and 6 months (6 M) post-ASDC, and ASDC-related complications within 6 M were evaluated in 184 adults: 79 with HTN (HTN+) and 105 without HTN (HTN−). Compared to HTN−, HTN+ patients were older and had a greater RV size and the prevalence of atrial arrhythmias, chronic heart failure, nonobstructive coronary artery disease, diabetes, hyperlipidemia, and left ventricular diastolic dysfunction. ASDC was successful and resulted in RVR, NoRS, and a lack of ASDC-related complications in the majority of HTN+ patients both at 24 h and 6 M. HTN+ and HTN− did not differ in ASD size, a successful implantation rate (98.7% vs. 99%), RVR 24 h (46.8% vs. 46.7%) and 6 M (59.4% vs. 67.9%) post-ASDC, NoRS 24 h (79% vs. 81.5%) and 6 M (76.6% vs. 86.9%) post-ASDC, and the composite of RVR and NoRS at 6 M (43.8% vs. 57.1%). Most ASDC-related complications in HTN+ occurred within 24 h and were minor; however, major complications such as device embolization within 24 h and mitral regurgitation within 6 M were observed. No differences between HTN+ and HTN− were observed in the total (12.7% vs. 9.5%) and major (5.1% vs. 4.8%) complications. Transcatheter ASDC is effective and safe in adults with secundum ASD and concomitant HTN in real-world clinical practice; however, proper preprocedural management and regular long-term follow-up post-ASDC are required.

## 1. Introduction

An ostium secundum atrial septal defect (ASD) is a common congenital heart defect in adults, which can result in an increased risk of heart failure (HF) and premature death [1,2,3,4,5]. The underlying hemodynamic disturbance in a secundum ASD is the presence of a left-to-right interatrial shunt, leading to a right ventricle (RV) volume overload and increased pulmonary flow volume, which over time can result in RV enlargement, pulmonary arterial hypertension (PAH), atrial arrhythmias, chronic HF, and thromboembolic complications [2,4,5,6,7,8]. Secundum ASD often remains undetected until adulthood, accounting for 25–30% of newly diagnosed congenital heart defects in adults [3,5].

Due to the ageing population, a number of patients with adult congenital heart disease (ACHD) continue to increase, including patients at an advanced age and/or with cardiovascular (CV) risk factors and acquired comorbidities, such as other CV diseases (CVDs) [9,10,11]. Systemic essential hypertension (HTN) remains a major CV risk factor, almost doubling the risk of death, with a rising systolic and diastolic blood pressure (BP) of as much as 20 and 10 mmHg, respectively [12,13]. The overall prevalence of HTN in the adult population is ~30–45% globally and ~43% in the Polish population [12,14]. HTN becomes progressively more common with an advancing age, reaching >60% in people aged >60 years [11,12,15]. Acquired comorbidities, such as HTN, are common in adults with ACHD, and can significantly contribute to their outcome [9,10,11]. HTN can lead to left ventricular (LV) hypertrophy (LVH), an abnormal LV compliance, and LV dysfunction, which may interfere with the disturbed hemodynamics associated with ASD [9]. As a result, concomitant HTN in adults with secundum ASD can aggravate hemodynamic disturbances, especially in older adults with a significant interatrial shunt and abnormal LV compliance.

Transcatheter ASD closure (ASDC) is the method of choice for treatment in adults with secundum ASD and a left-to-right interatrial shunt, which is recommended by the current guidelines regardless of patient age and symptoms [16,17,18]. Transcatheter ASDC is performed in the majority of adults with ASD, also commonly at an older age [9,16,17,18]. Several previous studies demonstrated the effectiveness of surgical and transcatheter ASDC in adults [19,20,21,22,23,24,25,26,27]. However, an unfavorable hemodynamic profile of adults with secundum ASD and concomitant HTN may contribute to a reduced efficacy and an increase in the risk of complications after transcatheter ASDC [16,17,18]. Data on effects of transcatheter ASDC in ASD adults with HTN remain limited [22,28,29,30,31]. While several previous studies of adults undergoing transcatheter ASDC included subpopulations of HTN patients, the effects of ASDC in ASD patients with HTN were not specifically addressed [22,26,28,29,30,31].

This study aims to evaluate the effectiveness and safety of transcatheter ASDC in adults with secundum ASD and concomitant systemic essential HTN in real-world clinical practice. The primary study endpoint is defined as a composite of a right ventricular (RV) reverse remodeling (RVR) and the lack of an interatrial residual shunt (NoRS) in echocardiography at 6 months (6 M) post-ASDC. The secondary study endpoint is the incidence of major ASDC-related complications within the 6 M follow-up.

## 2. Materials and Methods

### 2.1. Study Design and Patient Population

This longitudinal observational cohort study involved consecutive adult patients aged ≥18 years with secundum ASD who underwent an elective procedure of transcatheter ASDC at the Department of Cardiology and Internal Medicine, the University Hospital No. 1 in Bydgoszcz, Poland, from November 2004 to April 2016. Indication for ASDC was an isolated secundum ASD with a significant left-to-right interatrial shunt and signs of RV volume overload unless pulmonary vascular disease, irrespective of the presence of symptoms [16,17]. Documented paradoxical embolism was also considered as an indication for ASDC [16,17].

Patients with a suspicion or diagnosis of secundum ASD were referred to our center for transcatheter ASDC by their cardiologists. In our center, a specialized heart team— comprising of cardiologists with an expertise in general cardiology, ACHD, echocardiography, and interventional cardiology; a coordinator for transcatheter structural cardiac interventions, as well as a cardiothoracic surgeon and anesthesiologist if needed—was tasked with optimizing patient selection, procedural performance, echocardiographic guidance, and follow-up care. Prior to procedure, routine transthoracic echocardiography (TTE), transesophageal echocardiography (TEE), and cardiac catheterization if needed were performed. Routine coronary angiography was performed before ASDC in patients with symptoms of coronary artery disease (CAD) and all men aged >40 years and postmenopausal women regardless of symptoms to exclude obstructive CAD. After transcatheter ASDC, patients underwent serial follow-up examinations at 24 h and during elective follow-up hospitalization at 6 M as part of standard medical care at our center, including clinical examination, electrocardiography and TTE (at 24 h and 6 M), and TEE (at 6 M). Additionally, routine ambulatory TTE was recommended at 1 month and 3 months after ASDC. During 6 M follow-up hospitalization, particular care was taken to determine patient functional status and obtain information regarding symptoms development or any complications.

Clinical and echocardiographic data of patients undergoing transcatheter ASDC were collected prospectively for clinical purposes from November 2004 to December 2016 during three elective hospitalizations, which are part of standard medical care for patients undergoing transcatheter interventions at our center: (1) diagnostic hospitalization that aimed at confirming the diagnosis of significant secundum ASD and evaluating patient suitability for transcatheter ASDC; (2) intervention hospitalization that was performed within 1 month after diagnostic hospitalization in order to conduct ASDC procedure and included post-procedure evaluation of short-term ASDC effects and early ASDC-related complications; (3) follow-up hospitalization that was performed at 6 M post-ASDC for assessing ASDC effects at 6 M and late ASDC-related complications. Patient medical records were analyzed for baseline clinical characteristics, echocardiographic characteristics before and after ASDC, and clinical outcomes post-ASDC within 6 M follow-up.

The study was conducted in accordance with the Declaration of Helsinki. Approval from the Bioethics Committee of the Nicolaus Copernicus University in Toruń, Collegium Medicum in Bydgoszcz, Poland, was obtained (KB 449/2015). Data were collected prospectively for clinical purposes for all adults who underwent transcatheter ASDC and were followed for 6 months after the intervention within the period from November 2004 to December 2016. Retrospective analysis of data was performed for patients who underwent ASDC from November 2004 to May 2015. Written informed consent for the participation in the study was waived for this subgroup of patients given the retrospective nature of this analysis. All patients who underwent ASDC from June 2015 to April 2016 were subject to prospective observation for research purposes and provided informed consent for the participation in the study.

### 2.2. Echocardiography

Comprehensive two-dimensional (2DE) and Doppler echocardiography was performed using commercially available ultrasound instruments (Philips SONOS 7500, Philips Medical Systems, Bothell, WA, USA; Philips iE33, Philips Medical Systems, Bothell, WA, USA; GE Vivid q, GE Medical Systems, Milwaukee, WI, USA; GE Vivid S60, GE Medical Systems, Milwaukee, WI, USA; GE Vivid E9, GE Medical Systems, Milwaukee, WI, USA). All echocardiographic examinations were performed as part of standard medical care of adults undergoing diagnostics and treatment with transcatheter ASDC at our center [32]. All patients provided informed consent for TEE examinations.

Routine TTE and TEE were performed during the diagnostic hospitalization for evaluating ASD morphology, significance and suitability for transcatheter ASDC, and excluding other intracardiac lesions. During the intervention hospitalization, routine TEE was performed during the procedure for guiding procedure, assessing its direct effects, and excluding immediate ASDC-related complications. TTE was routinely performed at 24 h post-procedure for evaluating short-term effects of ASDC and excluding early ASDC-related complications. TEE at 24 h post-ASDC was only performed on indication (i.e., if clinical suspicion of complications occurred). The ASDC effects at 6 M and late ASDC-related complications were evaluated by routine TTE and TEE during the 6 M follow-up hospitalization. If a clinical suspicion of ASDC-related complications occurred at any time during a postintervention period, additional TTE and/or TEE were performed.

Baseline echocardiographic characteristics and changes in various echocardiographic parameters at 24 h and 6 M post-ASDC were evaluated. TTE and TEE were performed following the American Society of Echocardiography recommendations [33,34,35,36]. Echocardiographic examinations were performed by two cardiologists (I.Ś., M.W.) accredited at expert level in echocardiography in the referenced echocardiography lab. Echocardiographic recordings were analyzed independently by the same two cardiologists. The inter- and intra-observer coefficients of variation for all echocardiographic parameters were below 5.0% and below 2.5%, respectively. Measurements performed by both cardiologists from three consecutive cardiac cycles were averaged.

ASD diameter was measured by 2DE and color Doppler echocardiography (CDE) using TTE (from parasternal short axis, apical 4-chamber, and subcostal sagittal views) and TEE (from bicaval, aortic short-axis, and modified 4-chamber views). The maximum ASD diameter was obtained by 2DE (i.e., the maximum defect diameter) and CDE (i.e., the maximum jet width) using TTE and TEE.

Residual interatrial shunt was evaluated post-ASDC by CDE using standard TTE and TEE views for interatrial shunt imaging. The color scale settings were adjusted to optimize for the expected low velocity of shunting (i.e., 25–40 cm/s). NoRS was defined as the lack of interatrial left-to-right flow using CDE in both TTE and TEE. Small, moderate, and large residual interatrial shunts were defined as CDE jet width <2 mm, 2–4 mm, and >4 mm by TEE, respectively [37].

Measurements of RV end-diastolic (RVEDd), LV end-diastolic (LVEDd) and LV end-systolic (LVESd) diameters, LV wall thickness, and left atrial (LA) diameter were determined using TTE parasternal long axis view with M-mode cursor positioned just beyond the mitral leaflet tips, perpendicular to the LV long axis. Specifically, RVEDd was determined as the widest long-axis RV outflow tract diameter. An RVEDd of >27 mm was classified as enlarged RV [36]. RVR was defined as a relative decrease in RVEDd post-ASDC compared to baseline value. RVR was assessed 24 h (early RVR) and 6 M (late RVR) post-ASDC. LV remodeling (LVR) was defined as a relative increase in LVEDd at 6 M compared to baseline value. LV mass (LVM) was calculated according to Deveraux formula. LV ejection fraction (LVEF) was calculated by modified biplane Simpson’s method.

LV diastolic function was evaluated using the mitral valve (MV) inflow which was recorded by pulse-wave Doppler from TTE apical 4-chamber view. Peak E-wave (early transmitral flow) velocity and its deceleration time (DT) and peak A-wave (transmitral flow during atrial systole) velocity were measured. Mild and moderate LV diastolic dysfunction (LVDD) was defined as the presence of ratio of E/A < 0.8 and DT > 200 ms, while severe LVDD as E/A ≥ 0.8 and DT ≤ 200 ms [33].

Pulmonary artery systolic pressure (PASP) was estimated from RV systolic pressure using the tricuspid regurgitation (TR) jet velocity and the simplified Bernoulli equation, assuming that the normal peak RV systolic pressure should be less than 30–35 mmHg [35]. For the purpose of this analysis, PAH was defined as PASP ≥ 40 mmHg [35]. PASP was not calculated if TR was not present or the TR signal was inadequate to obtain reliable measurements. Pulmonary artery acceleration time (PAcT) was also measured [38]. Severity of TR and mitral regurgitation (MR) was evaluated following the recommendations.

### 2.3. Diagnosis of Systemic Essential Hypertension

Diagnosis of systemic essential HTN was determined by an attending cardiologist during hospitalization. Specifically, HTN was defined as systolic BP ≥ 140 mmHg and/or diastolic BP ≥ 90 mmHg as indicated by repeated BP measurements during hospitalization(s) or self-reported use of antihypertensive medication(s) due to prior HTN diagnosis [12]. BP levels were measured using a mercury sphygmomanometer and an appropriately sized BP cuff, i.e., the BP cuff covered 80% of the arm circumference in order to prevent a false under- or overestimation of BP readings. Readings were obtained after 5 min of seated rest. Three BP measurements were performed at least 1 to 2 min apart, and additional measurements only if the initial two readings differed by an amount greater than or equal to 10 mmHg. BP was then recorded as the average of the last two readings to define the systolic BP and diastolic BP levels. Out-of-office BP measurement was considered in patients with mildly or moderately elevated BP during diagnostic hospitalization (especially in those with grade 1 HTN) to provide supplementary clinical information for supporting the HTN diagnosis at intervention hospitalization.

In the group of patients with ASD and concomitant HTN (HTN+ group)—which was of primary interest to this study—the baseline clinical and echocardiographic characteristics, changes in echocardiographic parameters at 24 h and 6 M after ASDC, including occurrence of early and late RVR and interatrial shunting at 24 h and 6 M, as well as incidence of early and late complications were evaluated. In addition, comparisons of baseline clinical data, echocardiographic characteristics at three time points, and incidence of early and late ASDC-related complications between HTN+ group and group of ASD patients without HTN (HTN− group) were conducted for providing additional insights regarding effects of ASDC in real-world clinical practice.

### 2.4. Transcatheter Atrial Septal Defect Closure Procedure

Based on echocardiographic findings and clinical assessment, patients were considered eligible for transcatheter ASDC if they had: (1) an isolated secundum ASD with significant left-to-right interatrial shunt (typically native defect diameter >10 mm); (2) a maximum balloon-stretched defect diameter of ≤38 mm with a sufficient rim of ≥5 mm from the mitral and tricuspid valves except towards the aorta; (3) RV volume overload [16,17]. Patients were not considered eligible for transcatheter ASDC for the following reasons: the maximum native defect diameter >25 mm or balloon-stretched defect diameter of >38 mm, inadequate defect morphology (such as insufficient rim, multiple defects, or complex aneurysm), other types of ASDs (such as primum or sinus venosus), elevated PASP >80 mmHg, other hemodynamically significant intracardiac lesions (e.g., congenital cardiac defects such as anomalous pulmonary venous connection, significant mitral or aortic valve disease, etc.), RV dysfunction, and Eisenmenger physiology. In patients with a non-invasively estimated PASP of >50% of systemic pressure or >60 mmHg, cardiac catheterization for invasive evaluation was performed prior to intervention. Only patients with pulmonary vascular resistance of <5 Wood units either at baseline or after vasoreactivity testing with nitric oxide were considered for ASDC. In patients who were found to have an LA pressure of >15 mmHg during intraprocedural invasive evaluation, balloon occlusion of the ASD was performed and pressure measurement was repeated. In patients with an LA pressure increase of >10 mmHg, further HF treatment was requested before consideration for ASDC to reduce the risk of acute left HF after intervention.

Transcatheter ASDC procedure was carried out under local anesthesia and guided by fluoroscopy and TEE [32]. All patients had a device implanted using standard catheterization techniques by two interventional cardiologists. After hemodynamic assessment, all patients underwent “stop flow” balloon sizing of the defect. The device size chosen was 2–4 mm larger than the stretched ASD diameter. The following devices were used: Amplatzer Septal Occluder (St Jude Medical, St Paul, MN, USA), Figulla Flex Ⅱ ASD Occluder (Occlutech GmbH, Jena, Germany), and Ultrasept ASD Closure Device (Cardia, Inc., Eagan, MN, USA). Before device release, standard TEE views were used to confirm adequate device position. Procedural success was defined as successful implantation of the device without embolization or malposition with subsequent hospital discharge [39]. All patients provided informed consent for transcatheter ASDC procedure.

Intravenous heparin was administered during the procedure. Aspirin therapy (75 mg/day) was started 24 h before ASDC and continued for 6 months after the intervention. To prevent thrombus formation after device deployment, routine thromboprophylaxis with low-molecular weight heparin followed by warfarin was administered for 6 months unless it was contraindicated because of high bleeding risk. In those patients, dual antiplatelet therapy with aspirin 75 mg/day and clopidogrel 75 mg/day was administered. We note that our decision about the application of this antithrombotic treatment was primarily based on significant concerns associated with the device-related thrombosis risks and the lack of consensus recommendations on antithrombotic/antiplatelet treatment in patients undergoing transcatheter ASDC at the start of our study and during subsequent years. In addition, antibiotic prophylaxis for infective endocarditis was recommended during at least 6 months after ASDC procedure.

### 2.5. Transcatheter Atrial Septal Defect Closure-Related Complications

The incidence of early (within the first 24 h after the procedure) and late (in the period between 24 h and 6 M after the procedure) ASDC-related complications was evaluated based on clinical assessment, review of medical records, and TTE and TEE findings. Complications were also classified as total, major, and minor.

Early complications included: (1) major complications, such as death, cardiac arrest, stroke, peripheral embolism, device embolization or malposition needing surgery, cardiac perforation with tamponade, urgent surgical intervention, major bleeding, persistent arrhythmias, acute HF, endocarditis related to the device, and severe damage of femoral vessels, and echocardiographic findings, such as thrombus within the device, erosion or perforation of atrial wall or aorta, and acute MR or TR; (2) minor complications, such as pericardial effusion, minor venous access site bleeding or groin hematoma not requiring surgery, and transient arrhythmias [26,39]. Device embolization was defined as the movement of a device to a location other than atrial septum, whereas device malposition was defined as an unacceptable position of device within the atrial septum [39].

Late major complications included device-related death or sudden death, cardiac arrest, device embolization or malposition needing surgery, surgical reintervention, stroke, thromboembolic complications, endocarditis related to the device, and hospitalization due to exacerbation of HF, as well as echocardiographic findings at 6 M after ASDC, such as thrombus within the device, erosion or perforation of atrial wall or aorta, and a new onset or deterioration of MR. The deterioration of MR was defined as an increase in at least one grade after the procedure. Late minor complications were not evaluated due to limited data availability in patient medical records.

### 2.6. Primary and Secondary Study Endpoints

The primary study endpoint was defined as a composite of RVR and NoRS in echocardiography at 6 M post-ASDC for the HTN+ patients. The secondary study endpoint was an incidence of major ASDC-related complications within 6 M follow-up after ASDC procedure for the HTN+ patients.

### 2.7. Statistical Analysis

Statistical analyses were carried out using the Statistica 13.1 software (TIBCO Software Inc., Palo Alto, CA, USA). The Shapiro–Wilk test demonstrated non-normal distribution of the investigated data. Continuous variables were presented as medians with interquartile ranges. Comparisons of continuous variables between the groups of patients with and without HTN were performed by the Mann–Whitney unpaired rank sum test. Categorical variables were expressed as the number and the percentage. Categorical variables were compared using the χ2 test. Statistical significance was assumed at the level of *p* < 0.05.

## 3. Results

During the study period, 387 adults were hospitalized at our center for transcatheter structural cardiac interventions. Out of these, 333 patients were admitted with a suspicion or diagnosis of ASD. Among those patients, 208 patients were identified with a secundum ASD, including 194 patients considered as suitable candidates for transcatheter ASDC. The ASDC was not attempted in 10 patients for various reasons (see Section 3.3 for details). Those 10 patients underwent further evaluation by the heart team to identify the most appropriate treatment approach. Out of this group, eight patients had elective surgical ASDC. Out of the 184 patients with a median age of 47 (31–59) years, in whom transcatheter ASDC was attempted, 2 patients required urgent surgery due to device embolization that occurred before hospital discharge. As a result, a total of 182 patients underwent a successful transcatheter ASDC and were subject to the 6 M follow-up.

HTN was diagnosed in 79 patients (i.e., 42.9% of whole study population) out of 184 ASD patients undergoing transcatheter ASDC. Specifically, the diagnosis of HTN was determined in 75 patients during the diagnostic hospitalization based on the self-reported use of antihypertensive medication(s) due to prior HTN diagnosis. In addition, four patients were diagnosed with grade 2 HTN (systolic BP ≥ 160 mmHg and/or diastolic BP ≥ 100 mmHg) based on multiple BP measurements over several days of diagnostic hospitalization, which required the immediate initiation of an antihypertensive drug treatment. The ambulatory BP control post-intervention hospitalization was recommended in all HTN patients to achieve the BP targets [12].

HTN was more prevalent with the increasing age of the ASD patients. For example, HTN was diagnosed in 18% of patients aged <50 years and 74% of patients aged ≥50 years, and only in 3% of patients aged <25 years and 81% of patients aged ≥65 years.

### 3.1. Baseline Clinical Characteristics

The baseline clinical characteristics for the HTN+ and HTN− groups are displayed in Table 1. The HTN+ group comprised patients with a median age of 56 years and was characterized by the common prevalence of CV risk factors, such as hyperlipidemia and type 2 diabetes mellitus (T2D), and various comorbidities such as prior ischemic stroke, nonobstructive CAD, atrial arrhythmias and chronic symptomatic HF, as well as the frequent use of guideline-based pharmacotherapies such as beta blockers, angiotensin-converting enzyme inhibitors, angiotensin receptor blockers, and diuretics.

Compared to HTN+, HTN− patients were mostly younger, with a median age of 36 years. CV risk factors, such as hyperlipidemia and T2D, were more prevalent in the HTN+ group than HTN−. CAD was diagnosed only in HTN+ patients. A history of atrial arrhythmias was found more often in HTN+ than HTN− patients. Atrial fibrillation (AF) accounted for most atrial arrhythmias in both the HTN+ (90.5% of all arrhythmias) and HTN− (60%) groups. An AF, or atrial flutter, was found in an electrocardiogram performed before the procedure in 15.2% of the HTN+ patients vs. 2.9% of the HTN− patients (*p* = 0.003). Chronic symptomatic HF was more common in HTN+ than HTN− patients. In both the HTN+ and HTN− groups, almost all HF patients had symptoms of NYHA class ≤II (97.5% vs. 99%, *p* = 0.903). No difference in prior ischemic stroke was observed between groups. Compared to HTN−, HTN+ patients received guideline-based pharmacotherapies more frequently.

### 3.2. Baseline Echocardiographic Characteristics

The baseline echocardiographic characteristics for the HTN+ and HTN− groups are shown in Table 2. The HTN+ group was characterized by the presence of increased median values of RVEDd, LA diameter and LVM, as well as preserved LVEFand a common prevalence of RV enlargement, LVDD, PASP ≥ 40 mmHg, and significant TR.

An increased median RVEDd was found in both groups, but an increased median LA diameter only in the HTN+ group. The median RVEDd, LV and LA diameters, and LVM were larger in HTN+ compared to HTN− patients. RV enlargement was found in 91% of HTN+ patients vs. 79.5% of HTN− patients (*p* = 0.05). The median LVEF was preserved in both groups. At baseline, LVDD was observed more often in the HTN+ group than the HTN− one. While a mild and moderate LVDD was diagnosed in 25 HTN+ patients (42.4% of HTN+ group) and 8 HTN− patients (12.3% of HTN− group), severe LVDD was identified only in the HTN+ group (3 patients, 5.1% of HTN+ group). MR was moderate in all cases and more common in the HTN+ than the HTN− group.

No difference between the groups was found in the median maximum defect size; however, ASD was likely larger in the HTN− group than the HTN+ group. The median PASP was elevated in both groups; however, PASP ≥ 40 mmHg was more likely to occur in the HTN+ group than the HTN− group. While the median PAcT was shorter in the HTN+ group than in the HTN− group, it was >90 ms in both groups, indicating the lack of significant elevation in pulmonary vascular resistance [38]. Significant TR was observed in the majority of patients in both groups, but more often in the HTN+ group.

### 3.3. Transcatheter Atrial Septal Defect Closure Procedure

During the procedure, ASDC was not attempted in 10 patients for the following reasons: the balloon-stretched defect diameter was >38 mm (7 patients), ASD sinus venosus (1 patient), inadequate defect morphology (1 patient), and significant PAH (1 patient). As a result, ASDC was attempted in 184 patients (79 HTN+ patients and 105 HTN− patients) with an average balloon-stretched defect size of 19.7 ± 7.4 mm. A successful device implantation was performed in 182 patients, including 78 HTN+ patients (98.7% of HTN+ group) and 104 HTN− patients (99% of HTN− group, *p* for the difference between groups = 0.839). The two patients (one patient from the HTN− group and one HTN patient) had device embolization within 24 h after ASDC, requiring urgent surgery. The following types of devices were used for ASDC: the Amplatzer Septal Occluder in 43 HTN+ patients (54.4% of HTN+ group) and 51 HTN− patients (48.6% of HTN− group), the Figulla Flex Ⅱ ASD Occluder in 35 HTN+ patients (44.3%) and 50 HTN− patients (47.6%), and the Ultrasept ASD Closure Device in 1 HTN+ patient (1.3%) and 4 HTN− patients (3.8%).

### 3.4. Changes in Echocardiographic Parameters 24 h after Transcatheter Atrial Septal Defect Closure

Changes in echocardiographic parameters in the HTN+ group at 24 h after ASDC compared to the baseline values are presented in Table 3. Post-ASDC, the median RVEDd decreased. An early RVR occurred in 29 HTN+ patients (46.8% of HTN+ group). Post-intervention, the RV enlargement was found in 87.1% of the HTN+ patients. The median PASP, the incidence of PASP of ≥40 mmHg, and the occurrence of significant TR all decreased post-ASDC. An interatrial residual shunt was observed in 21% of HTN+ patients and was small in all patients. Neither severe MR nor intra-atrial thrombus were observed post-ASDC.

A comparison of the echocardiographic characteristics for the HTN+ and HTN− groups 24 h after ASDC is shown in Table 4. The median RVEDd decreased significantly in both groups (*p* = 0.003 for the HTN+ and *p* < 0.001 for the HTN− group). No difference in the incidence of early RVR was observed between groups. Post-ASDC, the median RV and LA diameters were greater in the HTN+ than the HTN− group. RV enlargement was found more often in the HTN+ group (87.1%) than in the HTN− group (72.8%) (*p* for the difference between groups = 0.03). A moderate MR was more frequent in HTN+ patients than the HTN− patients Neither severe MR nor intra-atrial thrombus were observed in both groups.

No difference was found between groups in the incidence of post-ASDC interatrial residual shunt, which was small in all patients. Post-intervention, the groups did not differ in the incidence of PASP ≥ 40 mmHg and median PASP, which was elevated in both groups. The incidence of significant TR decreased post-ASDC in both groups, but was higher in the HTN+ group.

### 3.5. Echocardiographic Characteristics 6 Months after Transcatheter Atrial Septal Defect Closure

Changes in the echocardiographic parameters in the HTN+ group at 6 months after ASDC compared to the baseline values are presented in Table 5. Compared to baseline, the median RVEDd decreased at 6 M post-ASDC. The late RVR was found in 38 HTN+ patients (59.4% of the HTN+ group). The RV enlargement was observed at 6 M in 84.4% of the HTN+ patients. The median LVEDd increased after 6 M. LVR occurred in 37 HTN+ patients (46.8% of the HTN+ group). Compared to baseline, LVDD was less common at 6 M, and no severe LVDD was found. While a moderate MR was less likely after 6 M post-ASDC, a new onset of a significant MR or deterioration of MR grade were observed in three HTN+ patients (4.7% of the HTN+ group). No device thrombosis was found; however, LA appendage thrombus was identified by TEE in two HTN+ patients (2.5% of the HTN+ group). An interatrial residual shunt was observed at 6 M in 15 HTN+ patients (23.4% of the HTN+ group), most of which were small; however, a moderate shunt was found in two patients (3.1% of the HTN+ group). Compared to baseline, the median PASP and incidence of PASP ≥ 40 mmHg and significant TR decreased after 6 M. The composite of RVR and NoRS was found at 6 M in 28 HTN+ patients (43.8% of the HTN+ group).

A comparison of the echocardiographic characteristics for the HTN+ and HTN− groups at 6 M after ASDC is shown in Table 6. Compared to baseline, the median RVEDd decreased post-ASDC in both groups (*p* < 0.001). No difference in the incidence of late RVR was observed between groups; however, the late RVR was more likely in HTN− patients. At 6 M post-ASDC, the median RV and LA diameters were larger in the HTN+ group compared to the HTN− group. The incidence of RV enlargement decreased after 6 M in both groups, but significantly only for HTN− patients (*p* = 0.002 for the HTN− vs. *p* = 0.244 for the HTN+ group). At 6 M, RV enlargement was found more frequently in the HTN+ group (84.4%) than the HTN− group (52.4%, *p* for the difference between groups < 0.0001). The median LVEDd increased after 6 M in both the HTN+ (*p* = 0.008) and HTN− (*p* < 0.0001) groups. No difference in LVR occurrence was observed between the HTN+ (46.8%) and HTN− group (44.2%*, p* for the difference between groups = 0.726).

Post-ASDC, LVDD was less common in both groups; however, LVDD (mild or moderate) occurred more frequently in the HTN+ than the HTN− group. No severe LVDD was found in either group. While MR was less likely in both groups after 6 M compared to baseline, it was more common in the HTN+ group. Additionally, a new onset of significant MR or deterioration of MR grade were found in 4.7% of the HTN+ group and 1.2% of the HTN− group at the 6 M echocardiography. While no intra-atrial thrombus was observed in the HTN− group at 6 M, LA appendage thrombus was identified by TEE in 2.5% of the HTN+ group. No device thrombosis was found in either group.

While an interatrial residual shunt was less likely to occur at 6 M post-ASDC in the HTN− group (13.1%) than the HTN+ group (23.4%), no significant difference was found between the groups. The residual shunt was mostly small in both groups, but a moderate shunt was found in 3.1% of the HTN+ and 2.4% of the HTN− group (two HTN− patients). Compared to baseline, the median PASP at 6 M was lower in both groups. The incidence of PASP ≥ 40 mmHg decreased after 6 M in both the HTN+ (*p* = 0.012) and the HTN− (*p* = 0.03) groups; however, PASP ≥ 40 mmHg was more common in the HTN+ than the HTN− group. Median PAcT increased post-ASDC in both groups, but more for the HTN− group. Compared to baseline, a significant TR was less common in both groups, but more for the HTN− group.

No difference in the composite of RVR and NoRS was observed at 6 M post-ASDC between the groups; however, the concurrent occurrence of RVR and NoRS was more likely in the HTN− group compared to the HTN+ group.

### 3.6. Transcatheter Atrial Septal Defect Closure-Related Complications

ASDC-related complications for the HTN+ and HTN− groups within 6 M after ASDC are shown in Table 7. There were no deaths during the 6 M follow-up in the studied population. No significant differences in the incidence of total and major, as well as early and late, ASDC-related complications were observed between groups.

Most complications occurred within 24 h post-ASDC in both the HTN+ (70% of all complications in this group) and HTN− (80%) group. The majority of early complications in both the HTN+ (85.7% of all early complications) and HTN− (62.5%) were minor, comprising mostly of minor bleedings associated with the venous access site. Early major complications occurred in one HTN+ patient (1.3% of the HTN+ group) and three HTN− patients (2.9% of the HTN− group). Specifically, in the HTN+ group, there was one Figulla Flex Ⅱ ASD Occluder device embolization detected by routine TTE at 24 h after procedure. In the HTN− group, the following early major complications were observed: an Ultrasept ASD Closure Device embolization during the procedure, cardiac arrest due to a slow junctional rhythm followed by asystolia during the procedure with a successful resuscitation (without the recurrence of bradyarrhythmia and need for a pacemaker implantation in the follow-up), and HF exacerbation immediately after the procedure requiring parenteral diuretic. Both patients with device embolization underwent a successful urgent surgical intervention.

Late major complications in the HTN+ group included a new onset of a moderate-to-severe MR in one patient and the deterioration of MR (from moderate to moderate-to severe MR) in two patients. In the HTN− group, there was one case of the deterioration of MR (from moderate to severe MR) and one hospitalization for HF exacerbation within 6 M.

At the 6 M follow-up, the incidence of symptomatic HF was 32.1% in the HTN+ group vs. 10.6% in the HTN− group (*p* < 0.001). All HF patients from the HTN+ group and 90.9% of HF patients from the HTN− group had HF symptoms of NYHA class ≤II.

### 3.7. Primary and Secondary Study Endpoints

A composite of RVR and NoRS in echocardiography at 6 M post-transcatheter ASDC, which was the primary study endpoint, occurred in 43.8% of the HTN+ group. The incidence of major ASDC-related complications within the 6 M follow-up after the transcatheter ASDC procedure, which was the secondary study endpoint, occurred in 5.1% of the HTN+ group.

## 4. Discussion

The present study is a unique comprehensive analysis of the effectiveness of transcatheter ASDC in a large, unselected cohort of adults with secundum ASD and concomitant HTN at intervention. Our findings confirmed the feasibility, effectiveness, and safety of transcatheter ASDC in a high-risk population of adults with HTN in real-world clinical practice. Our study provides evidence that transcatheter ASDC can be successfully performed and is effective in adults with HTN, and also in symptomatic and older patients. Based on our findings, the abolishment of an interatrial shunt, regression of RV size, decrease in PASP, and clinical improvement can be expected post-transcatheter ASDC in the majority of adults with HTN. In addition, our results demonstrate that transcatheter ASDC in patients with HTN is technically easy, safe, and associated with a low rate of complications comparable to younger adults without HTN. Given a progression of RV enlargement, an elevation in PASP, the development of LVH and LVDD, and escalation of symptoms with increasing age, transcatheter ASDC can be recommended in adults with diagnosed secundum ASD and concomitant HTN. However, an in-depth preprocedural evaluation and proper management, including the use of guideline-based pharmacotherapies, as well as regular long-term follow-up visits after the procedure are required in adults with HTN for improving ASDC effectiveness and reducing the risk of complications.

The main findings of our study are: (1) among adults with secundum ASD undergoing transcatheter ASDC in real-world clinical practice, 43% had systemic essential HTN with a common occurrence of LVDD (48% of HTN patients), chronic HF (38%), MR (36%), atrial arrhythmias (27%), prior ischemic stroke (17%), nonobstructive CAD (8%), and CV risk factors such as T2D (15%) and hyperlipidemia (49%), all of which were more prevalent in patients with HTN compared to those without; (2) ASD patients with HTN had a median age of 56 years, which was higher than that for patients without HTN (36 years); (3) ASD patients with HTN commonly received guideline-based pharmacotherapies before ASDC, and more often than patients without HTN; (4) the successful device implantation rate in HTN patients was high and comparable to those without HTN (99%); (5) in HTN patients, RVR was observed post-ASDC in 47% of patients at 24 h and 59% at 6 M, whereas NoRS was observed in 79% and 77%, respectively; these statistics were comparable to patients without HTN; (6) patients with and without HTN did not differ in the composite of RVR and NoRS at 6 M post-ASDC; however, the concurrent occurrence of RVR and NoRS was more likely in patients without HTN (57%) than with HTN (44%); (7) the prevalence of PASP ≥ 40 mmHg and significant TR decreased post-ASDC in HTN patients, but less than in those without HTN; (8) the rates of total and major ASDC-related complications were low in HTN patients and comparable to those without HTN; (9) most ASDC-related complications in HTN patients occurred within 24 h and were minor; however, 5% of the patients had major complications, such as device embolization within 24 h and a new onset or deterioration of MR within 6 M.

Transcatheter ASDC is recommended in adults with secundum ASD, if an RV enlargement and suitable defect morphology are confirmed, regardless of the symptoms and patient age [16,17,18]. The transcatheter ASDC approach has become an accepted alternative to surgical repair due to decreased complication rates, shorter hospital stays, and greater cost-effectiveness [22,24,26]. Patients’ morbidity seems to benefit from ASDC at any age, especially if a transcatheter closure is performed [18,21,23,25,27,28,29]. Additionally, the results of a few cohort studies demonstrated improvements in the RV size, PASP and functional status, and long-term mortality that was not inferior to surgical treatment [21,23,24,28].

However, the indications for ASDC in specific subpopulations of adults can be ambiguous. While patients with secundum ASD frequently remain asymptomatic until adulthood, most of them develop symptoms beyond the fourth decade, and have a reduced life expectancy compared to the general population [2,18,31]. A lower relative mortality of ASD adults undergoing a surgical or transcatheter closure compared to those not undergoing ASDC was observed in the follow-up that lasted up to 53 years [31]. In addition, perioperative and mid to longer-term survival in a large contemporary adult cohort undergoing ASDC, catheter or surgical, was shown to be excellent irrespective of the age, gender, and mode of closure, and was similar when matched to the general population [40]. Additionally, a weak protective effect of transcatheter ASDC on the adjusted mortality rate was observed in patients aged >50 years, but there was no significant impact of closure on atrial arrhythmias [23]. However, the selection of candidates for transcatheter ASDC who are aged >40–60 years and have various comorbidities, especially if they are asymptomatic or mildly symptomatic, can be controversial, as their outcome may be inferior compared to that of younger adults without comorbidities [18,41]. Evidence on the effects of transcatheter ASDC in adults with secundum ASD and other concomitant CVDs is notably limited [22,23,24,25,26,27,28,29,30]. Several previous studies examined adult populations undergoing transcatheter ASDC; however, these studies did not elucidate the effectiveness and safety over a long-term period in adults with concomitant systemic essential HTN [22,26,28,29,30]. In addition, some of these studies had limitations associated with heterogeneous populations (e.g., enrolling patients not only with secundum ASD, or including both children and adults), a small sample size, and the lack of well-defined endpoints, an echocardiographic assessment at multiple time-points post-ASDC, as well as a long-term follow-up [22,23,24,25,42].

Adults with secundum ASD, especially older, can present a higher morbidity and mortality associated with the late presentation of ASD and an increased prevalence of comorbidities with advancing age. The prevalence of HTN in patients with ACHD was found to be 30–60% [9,10,11,41]. Comorbidities such as HTN and vascular disease were mainly present in patients older than 40 years [9,10,11,28,41]. Previous studies of adults undergoing transcatheter ASDC reported the prevalence of HTN in 4–12% in patients aged <30 years [22,26,31], 12–25% in patients aged 40–60 years [28,29], and 25–61% in patients >60 years old [11,27,28,30]. In our study, 43% of adults with secundum ASD at the median age of 47 years had concomitant HTN. Based on our findings, HTN is common in adults with secundum ASD in real-world clinical practice, especially in older populations, and its specific impact on the hemodynamics and clinical outcome should be recognized and considered in patient management. In addition, in adults with ASD and concomitant HTN, we observed a common prevalence of CV risk factors, such as hyperlipidemia and T2D, as well as atherosclerotic CVD such as CAD and stroke, which were at least as common as in previous studies reporting the incidence of T2D in the range 1–14% [22,26,27,28,29,30,31], prior ischemic stroke 1–8% [22,26,27,29,31], and CAD 2–14% [22,27,28,30,31]. Concomitant CV risk factors or other CVDs could affect hemodynamics, clinical status, and outcome of adults with ASD [9,10,11,43].

Natural history studies of patients with unrepaired ASD showed that the RV volume overload caused by an interatrial shunt is common, and tend to increase progressively over time, thereby affecting cardiac performance even in asymptomatic patients [2,22]. Unrepaired ASDs also result in a progressive elevation in PASP with increasing age [2,6,8,28]. In our study of ASD adults at the median age of 47 years, RV enlargement was observed in 85% of patients, more frequently in HTN patients compared to those without HTN. Additionally, the median RV size was greater and elevated PASP more likely in HTN patients compared to those without HTN. Previous studies demonstrated that the RV size and PASP correlated weakly with the defect size; however, in our study, the ASD size was more likely smaller in patients with HTN than without [28].

We observed a decrease in the RV size 24 h post-transcatheter ASDC, with further reduction during the following 6 months. While RVR in the 6 M follow-up occurred in 68% of adults without HTN, RVR was also observed in the majority of HTN patients (59%), although the latter were older and presented with a larger RV before and after the intervention, and more often had significant TR. The lack of severe PAH in HTN patients in our study could facilitate RVR post-ASDC [28]. However, patients with a more advanced age and common LVDD, which could result in a higher LA pressure and greater interatrial shunting volume, could contribute to the long-standing and substantial RV volume overload and less effective RVR post-ASDC in HTN patients, compared to those without HTN in our study [27,28,44,45]. ASDC was previously shown to result in a rapid reduction in RV volume progressing over the two years after closure, especially in patients aged <50 years [20,23,28,29,42,44,45,46,47,48,49,50,51,52,53]. However, some patients, mostly older than 40 years, were found to have persistent RV enlargement post-ASDC in a multi-year observation, which suggested an impaired ability of RV to remodel due to prolonged volume overload or ageing-related decreased compliance [23,54,55]. Our findings indicate that despite ongoing RVR post-ASDC, the majority of HTN patients had persistent RV enlargement at 6 months post-intervention.

Residual interatrial shunting post-ASDC, which was observed in 23% of HTN patients after 6 months in our study, could potentially unfavorably affect RVR in the long-term follow-up. However, an incidence of residual shunting was not significantly higher in HTN patients compared to those without HTN. In addition, residual shunts were mostly small, with only a 3% incidence of moderate shunting among HTN patients. Additionally, a relationship between the incidence and size of residual shunting and the persistence of RV enlargement post-ASDC has not been established [54,55]. The incidence of residual shunting post-transcatheter ASDC in adults was previously reported to have been 4% [56], 6% [57], 8% [58], 9% [26,30,59], 18% [22], and 50% [54], depending on the criteria of shunt diagnosis, patient age, the time point of assessment, type of device, and use of TTE or TEE for shunt detection. Residual shunts were detected less frequently with an increasing time elapsed since ASDC (e.g., in 6–9% of patients at the follow-up of 2.3–3 years) [56,57,60]. In addition, while some authors reported at least moderate shunting only, in our analysis, we included all residual shunts detected by TEE, even trivial, which could also affect the prevalence of 23% in HTN patients in our study [28,37]. The incidence of 3% of moderate shunting 6 months post-ASDC in our study was comparable to other results [22,58]. Our findings indicate that the late presentation of ASD, older age, greater prevalence of LVH and LVDD, as well as post-ASDC increase in the LV preload and LA pressure may contribute to the presence and persistence of residual interatrial shunts post-ASDC in adults with HTN.

We observed a reduction in PASP post-ASDC; however, a persistent elevation in PASP was found in some patients, more often in those with HTN than without. Previous evidence showed that transcatheter ASDC led to an immediate decrease in PASP, followed by a progressive normalizing trend in the long-term follow-up, especially in younger patients and those with moderately elevated PASP, but also in patients aged >60 years [5,23,28,56]. However, some patients (e.g., 29% in [54], 51% in [28], as well as 31% of patients without HTN and 53% of HTN patients in our study) demonstrated the persistent elevation of PASP post-ASDC at rest or during exercise, especially those aged >40–60 years and patients with concomitant HTN [28,54,56]. These findings suggest that a proportion of adults with ASD, especially among those with significantly elevated PASP, have permanently elevated pulmonary vascular resistance, which may unfavorably affect improvements in PASP and clinical symptoms after ASDC [28,54,61]. Older age, the late presentation of ASD, long-standing RV volume overload, and common LVDD could contribute to smaller improvement in PASP post-ASDC in adults with HTN than those without HTN in our study.

In our study, the LA and LV size in adults with HTN exhibited recognizable remodeling at 6 months after transcatheter ASDC, which was also observed in other studies [29,46]. In addition, we found a decrease in the prevalence of LVDD after ASDC in HTN adults. The reports on interactions between RV and LV in secundum ASD indicated a normalization of abnormal septal motion and septal curvature at the end diastole with an improvement in the RV function after ASDC, which can be an important benefit of intervention, particularly for adults at older age or those with HTN [5,23,28,50,51].

Functional capacity in the majority of adults with unrepaired secundum ASD is substantially impaired and the severity of functional limitation increases with advancing age [2,62]. This is likely a reflection of the ASD-related increased pulmonary blood flow and PAH worsened by impaired LV compliance as a result of RV volume overload, increasing age, and the common presence of other CVDs [23,28,63,64]. A reduction in LV compliance, presence of LVH, development of significant LVDD, as well as LA enlargement and elevation of LA pressure associated with comorbidities, such as HTN or CAD, can increase a left-to-right interatrial shunt and worsen hemodynamic disturbances and clinical symptoms in adults with ASD and other concomitant CVDs [4,18]. The incidence of HF among patients with secundum ASD was reported as 1–3% in patients aged 10–29 years [22,26], 54% at the age of 49 years [28], and 87% at the age of 71 years [28]. We demonstrated that symptomatic HF, LVDD, MR, and atrial arrhythmias were common and occurred more often in adults with ASD and concomitant HTN compared to those without HTN, which is consistent with the results of ASD studies with a high prevalence of HTN [28,29,30]. A lower prevalence of symptomatic HF in our study compared to the study of Humenberger et al. [28] (23% vs. 51% of patients) despite a higher prevalence of HTN in our study, could result from a smaller defect size and smaller RV enlargement in our population. In addition, most HTN patients in our study received guideline-based pharmacotherapies for HTN and/or HF symptoms.

We observed a decrease in the incidence of symptomatic HF in HTN patients at 6 months post-ASDC. The abolishment of interatrial shunt and substantial RVR, followed by LVR and an increased LV stroke volume, could contribute to improving HF symptoms and the functional status of HTN patients post-ASDC in our study [20,29,30,65,66]. It was previously shown that surgical ASDC prevented functional deterioration due to HF, especially in young adults [20]. Additionally, several studies and pooled analyses demonstrated benefits of transcatheter ASDC on functional capacity in adults at any age, both asymptomatic and with a diminished exercise capacity and reduced cardiopulmonary function; however, the best outcome was observed in patients with less functional impairment and less elevated PASP [23,25,27,28,29,30,44,45,58,65]. Additionally, a time delay in the improvement of exercise capacity compared with cardiac remodeling was observed [29].

Our study demonstrates that transcatheter ASDC in adults with HTN, and also in symptomatic and older patients, is technically easy, safe, and results in a low rate of complications comparable to younger patients without HTN. A successful device implantation rate of 99% in our study was similar to other reports [28,56,67]. An incidence of 13% of the total ASDC-related complications during the 6 M follow-up in HTN adults in our study was comparable to patients without HTN. Previous studies of adults undergoing transcatheter ASDC indicated an incidence of complications of 2–15%, depending on patients’ age, type of device, length of follow-up, and criteria for complications [22,24,26,30,39,56,67,68,69,70]. In our study, most complications in adults with HTN were minor and occurred within 24 h after ASDC. An incidence of 9% of early complications in HTN patients was comparable to patients without HTN in our study and results of other studies [26,39,68,69]. A high prevalence of chronic antithrombotic therapy in HTN patients, mostly for thromboembolic prevention in AF, could have contributed to the occurrence of early minor bleedings associated with the venous access site in our study; however, no major bleedings were observed in contrast to other studies [69]. In the study by Butera et al. [26], minor complications were independently related to the presence of systemic HTN.

The incidence of 5% of major complications in HTN adults in our study was comparable to that in patients without HTN, as well as to the results of previous studies [22,24,26,39,56,67,68,69]. In our study, no case of death occurred in the whole study group within the 6 M follow-up after intervention. The only early serious complication in HTN adults was device embolization within 24 h post-ASDC, which also occurred in one patient without HTN. Both cases of device embolization occurred only during the very initial phase of transcatheter structural cardiac interventions at our center, which may indicate the importance of an increased operator’s experience over time with significant implications for patient safety [71]. The device embolization was observed at a similar frequency and reported as the most common early ASDC-related complication in other studies [18,22,26,39,56,57,58,67,68,69]. In the following 6 months post-ASDC, we did not observe any serious complications in the HTN adults, such as device migration, reintervention, HF exacerbation or a new onset of HF, endocarditis, erosion of LA or aorta wall, device thrombosis, thromboembolic events, or ischemic stroke, which were reported in previous studies with a longer observation period of 18 months to 5 years [24,39,69,72,73]. However, a new onset of significant MR and the deterioration of MR were observed within 6 M post-ASDC in three HTN patients (4% of HTN group) compared to one patient without HTN (1%).

It appears that the occurrence of MR should not be neglected in the analysis of ASDC-related complications, especially as it was also observed by other authors in 4–11% of patients in the period between 1 month and 6 years post-transcatheter ASDC [63,74,75,76,77]. Based on previous studies, mainly on surgical ASDC, it has been generally believed that mild MR does not progress or even improve with ASD repair due to the restoration of septal configuration [20,59,74,75]. However, despite a decrease in volume overload of pulmonary circulation, MR may develop or deteriorate after ASDC due to an increased LV preload, LA and LV remodeling, the dilation of mitral annulus, and diminished coaptation of MV leaflets, especially in older adults with degenerative MV changes [20,59,74,75]. In addition, older age, the female gender, presence of AF, and large interatrial shunt volume were found to be associated with a risk of significant MR post-ASDC [74,75,76,77]. Moreover, patients with ASD and severe MR may not manifest symptoms of MR before ASDC due to the unloading of LA by the presence of ASD butmay develop symptoms of pulmonary venous hypertension post-ASDC [63]. In most cases, an improvement in hemodynamics caused by ASDC may be more important for clinical outcomes in a long-term follow-up, rather than MR deterioration [77]. However, given a risk of MR deterioration following ASDC in adults in the mid to long-term, MV annuloplasty with a surgical ASD repair should be considered, and a long-term follow-up post-ASDC is recommended in adults with pre-closure MR, especially in elderly patients with risk factors such as a large interatrial shunt, AF, or PAH [74,75,76]. Older age, LA and LV remodeling, and a relatively common occurrence of AF could have contributed to the developing and deteriorating MR in HTN patients in our study; however, the prevalence of this complication was very low.

Despite advances in transcatheter ASDC, post-closure HF in adults in short-term and long-term follow-ups remains a clinical problem, especially in older adults and those with other concomitant CVD [29,56,70,78,79,80]. In HTN patients, ASDC can be associated with an acute HF or worsening chronic HF [18,48,66,79,80,81,82]. The potential mechanism of HF post-ASDC in HTN patients can be an increased LV preload in the setting of thereduced LV compliance due to RV enlargement and altered interventricular interaction, as well as the presence of LVDD associated with HTN [66,70,79,80,81]. In older adults, the LV diastolic function can also be affected by ageing through several mechanisms, such as ventricular and vascular stiffening. In addition, LVDD may progress after ASDC in older patients, thereby further increasing the LV filling pressure [48]. Additionally, the presence of left-to-right interatrial shunt may mask LVDD before ASDC [70,80,82]. An assessment of the pre-closure LV diastolic function, including the testASD occlusion, is recommended in identifying high-risk patients for post-closure HF, who may require anti-HTN and anti-HF therapy or pharmacologic preconditioning before ASDC, or the use of a fenestrated device [18,79,80,83]. In our study, the common administration of guideline-based pharmacotherapies for HTN and/or HF before ASDC appeared to be effective for preventing post-closure HF in HTN patients. It is to be noted that the two patients without HTN, who developed post-closure HF, had LVDD, and one of them who was asymptomatic before ASDC did not receive anti-HF therapy.

Through various mechanisms (e.g., LVH, decreased LV compliance, LVDD, AF, and elevated LA pressure), HTN can unfavorably affect ASD-related disturbances in hemodynamics, leading to a larger interatrial shunting volume, greater RV volume overload, the development of RV dysfunction, and more severe PAH, all of which can contribute to worse effects of ASDC in adults with secundum ASD and HTN, regardless of the patient’s age. In our real-world study, the older age of hypertensive ASD patients was likely a factor affecting the ASDC results. It is essential to emphasize that this is a factor present in real-world clinical practice because adults with secundum ASD and HTN are generally relatively old and non-hypertensive ASD adults are relatively young. For example, in our study, HTN was diagnosed in only 3% of patients aged <30 years compared to 81% of patients aged ≥65 years. The majority of ASD patients with HTN was aged ≥50 years, and most non-hypertensive patients were aged <50 years.

This study aimed to explore real-world data on the effectiveness and safety of transcatheter ASDC in adults with secundum ASD and concomitant systemic essential HTN. Given the ageing population, the growing prevalence of HTN in adults (especially older), and broadening indications for transcatheter structural cardiac interventions in patients with ACHD, we view our findings to provide insights regarding both the mechanisms of the effects of transcatheter ASDC and clinical implications related to the effectiveness and safety of transcatheter ASDC in adults in real-world clinical practice. While our results support transcatheter ASDC for the treatment of hypertensive adults, there is a continued need for further clinical research, including multi-center studies with a larger sample size, to optimize the management and elucidate the effectiveness and safety of transcatheter ASDC in adults with HTN in a multi-year follow-up. In addition, improvements in ACHD care management are needed [84,85].

Our study included the analysis of a comprehensive set of various data that were prospectively collected over the period of about 12 years, a thorough review of medical records, well-defined study outcomes, and a relatively long-term follow-up. Our approach was focused on evaluating the outcomes in adults with secundum ASD and HTN who, in real-world clinical practice, are generally relatively old. However, the results for non-hypertensive adults undergoing ASDC who, in real-world clinical practice, are relatively young were also provided. In our study, about 75% of ASD patients with HTN had an age of >50 years and about 75% of patients without HTN had an age of <50 years (Table 1). As a consequence, the groups with and without HTN were highly divergent in terms of age, so an attempt to perform an analysis after age matching would unavoidably omit a large portion of patients. As our work aimed to present real-world clinical data, the analysis of the limited subpopulation of patients was not desirable. It is also to be noted that further generalization of our findings requires caution associated with the 6 M follow-up period, and the use of a single RV dimension for the RVR assessment because it was the only RV parameter available in the majority of patients throughout the period of the study.

## 5. Conclusions

Systemic essential HTN is a common comorbidity in adults with secundum ASD in real-world clinical practice. The group of patients with ASD and HTN and the median age of 56 years had various acquired comorbidities including other types of CVDs and common CV risk factors. Our findings support the transcatheter ASDC approach for adults with secundum ASD and concomitant HTN. Transcatheter ASDC was feasible, effective, and safe in adults with HTN. Transcatheter ASDC in HTN adults resulted in an abolishment of interatrial shunt and a decrease in the RV size in the majority of patients, and was associated with a low rate of complications that were mostly minor. However, in adults with secundum ASD and concomitant HTN, special attention should be given to provide an in-depth preprocedural evaluation and management, and conduct regular long-term follow-ups post-ASDC to improve the clinical outcome and reduce complications.

## Figures and Tables

**Table 1:** Baseline clinical characteristics of patients with secundum atrial septal defect and with (HTN+ group) or without (HTN− group) systemic essential hypertension.

Variable	HTN+ Group (*n* = 79)	HTN− Group (*n* = 105)	*p*-Value between Groups
Age (years)	56.2 (51.0–62.0)	36.1 (25.0–47.0)	<0.0001
Gender (male/female) *n* (%)	25/54 (31.6/68.4)	29/76 (27.6/72.4)	0.485
Smoking *n* (%)	6 (7.6)	7 (6.7)	0.822
Hyperlipidemia *n* (%)	39 (49.4)	23 (21.9)	0.0001
Nonobstructive coronary artery disease *n* (%)	6 (7.6)	0 (0.0)	0.004
Ischemic stroke *n* (%)	13 (16.5)	14 (13.3)	0.572
Type 2 diabetes mellitus *n* (%)	12 (15.2)	0 (0.0)	<0.001
Chronic symptomatic heart failure *n* (%)	30 (38.0)	13 (12.4)	<0.001
Atrial arrhythmias *n* (%)	21 (26.6)	10 (9.5)	0.003
Atrial fibrillation paroxysmal/permanent *n* (%)	8/11 (10.1/13.9)	3/3 (2.9/2.9)	0.003
ASA *n* (%)	30 (38.0)	24 (23.1)	0.029
Beta blocker *n* (%)	57 (72.2)	22 (22.9)	<0.00001
ACEI or ARB *n* (%)	35 (44.3)	5 (4.8)	<0.00001
Diuretic *n* (%)	18 (31.6)	3 (2.9)	<0.00001
Antithrombotic treatment *n* (%)	25 (31.7)	5 (4.8)	<0.00001
Coronary angiography before ASDC *n* (%)	24 (30.4)	12 (11.4)	0.001

Data represent the number of patients (*n*), including the percentage of total number (%) or median values with corresponding interquartile range (in parenthesis). Abbreviations: ACEI—angiotensin-converting enzyme inhibitor; ARB—angiotensin receptor blocker; ASA—acetylsalicylic acid; ASDC—transcatheter atrial septal defect closure; HTN+—group of patients with essential hypertension; HTN−—group of patients without essential hypertension.

**Table 2:** Baseline echocardiographic characteristics of patients with secundum atrial septal defect and with (HTN+ group) or without (HTN− group) systemic essential hypertension.

Variable	HTN+ Group (*n* = 79)	HTN− Group (*n* = 105)	*p*-Value between Groups
RVEDd (mm)	33.0 (30.0–39.0)	31.0 (28.0–37.0)	0.028
LA (mm)	41.5 (37.0–45.0)	35.0 (31.0–38.0)	<0.0001
LVEDd (mm)	44.0 (41.0–48.0)	42.0 (39.0–46.0)	0.014
LVESd (mm)	30.0 (25.0–33.0)	27.0 (23.0–31.5)	0.02
IVSd (mm)	11.0 (10.0–12.0)	9.0 (8.0–11.0)	<0.0001
PWd (mm)	10.0 (10.0–11.0)	8.0 (7.0–10.0)	<0.0001
LVM (g/m^2^)	158.0 (132.0–206.0)	110.0 (82.0–170.0)	<0.0001
LVEF (%)	60.0 (55.0–60.0)	60.0 (60.0–64.0)	0.004
LVDD ^a^ *n* (%)	28 (47.5)	8 (12.3)	<0.0001
MR ^b^ moderate *n* (%)	24 (35.8)	13 (15.7)	0.004
Maximum defect native diameter (mm)	11.0 (6.0–18.0)	15.0 (7.0–20.0)	0.386
PASP ^b^ (mmHg)	46.0 (40.5–52.0)	44.0 (39.0–48.0)	0.120
PASP ≥ 40 mmHg ^b^ *n* (%)	52 (77.6)	55 (66.3)	0.127
PAcT ^b^ (ms)	96.0 (74.0–110.0)	107.0 (89.0–120.0)	0.017
TR ^b^ moderate or severe *n* (%)	54 (80.6)	48 (57.8)	0.001

Data represent the number of patients (*n*), including the percentage of total number (%) or median values with corresponding interquartile range (in parenthesis). Abbreviations: IVSd—intraventricular septum wall thickness; LA—left atrial diameter; LVDD—left ventricular diastolic dysfunction; LVEDd—left ventricular end-diastolic diameter; LVEF—left ventricular ejection fraction; LVESd—left ventricular end-systolic diameter; LVM—left ventricular mass; MR—mitral regurgitation; PAcT—pulmonary artery acceleration time; PASP—pulmonary artery systolic pressure; PWd—left ventricular posterior wall thickness; RVEDd—right ventricular end-diastolic diameter; TR—tricuspid regurgitation. ^a^ Data were available for 59 patients from HTN+ group and 65 patients from HTN− group. ^b^ Data were available for 67 patients from HTN+ group and 83 patients from HTN− group.

**Table 3:** Changes in echocardiographic parameters 24 h after transcatheter atrial septal defect closure (ASDC) compared to baseline values in the group of patients with systemic essential hypertension (HTN+).

Variable	Before ASDC (*n* = 79)	24 h after ASDC (*n* = 79)	*p*-Value
RVEDd (mm)	33.0 (30.0–39.0)	32.0 (28.0–38.0) ^a^	0.003
LA (mm)	41.5 (37.0–45.0)	42.0 (38.5–44.5)	0.668
LVEDd (mm)	44.0 (41.0–48.0)	42.5 (36.0–49.0)	0.009
LVESd (mm)	30.0 (25.0–33.0)	30.0 (25.0–33.0)	0.055
LVEF (%)	60.0 (55.0–60.0)	60.0 (55.0–60.0)	0.258
MR moderate *n* (%)	24 (35.8) ^b^	20 (32.3) ^a^	0.670
PASP (mmHg)	46.0 (40.5–52.0) ^b^	40.0 (39.0–47.0) ^b^	0.01
PASP ≥ 40 mmHg *n* (%)	52 (77.6) ^b^	34 (50.7) ^b^	0.012
PAcT (ms)	96.0 (74.0–110.0) ^b^	104.0 (96.0–133.0) ^b^	0.168
TR moderate or severe *n* (%)	54 (80.6) ^b^	34 (50.7) ^b^	<0.001

Data represent the number of patients (*n*), including the percentage of total number (%) or median values with corresponding interquartile range (in parenthesis). Abbreviations: ASDC—atrial septal defect closure; LA—left atrial diameter; LVEDd—left ventricular end-diastolic diameter; LVEF—left ventricular ejection fraction; LVESd—left ventricular end-systolic diameter; MR—mitral regurgitation; PAcT—pulmonary artery acceleration time; PASP—pulmonary artery systolic pressure; RVEDd—right ventricular end-diastolic diameter; TR—tricuspid regurgitation. ^a^ Data were available for 62 patients. ^b^ Data were available for 67 patients.

**Table 4:** Comparison of echocardiographic characteristics of patients with secundum atrial septal defect and with (HTN+ group) or without (HTN− group) systemic essential hypertension at 24 h after transcatheter atrial septal defect closure (ASDC).

Variable	HTN+ Group (*n* = 79)	HTN− Group (*n* = 104)	*p*-Value between Groups
RVEDd ^a^ (mm)	32.0 (28.0–38.0)	29.0 (26.0–33.0)	0.013
Relative RVEDd change compared to baseline ^a^ (mm)	−1.0 (−5.0–−1.0)	−2.0 (−4.0–−1.0)	0.551
Reverse RV remodeling ^a^ *n* (%)	29 (46.8)	43 (46.7)	0.997
LA (mm)	42.0 (38.5–44.5)	35.0 (32.0–38.0)	<0.0001
LVEDd (mm)	42.5 (36.0–49.0)	43.0 (39.0–47.0)	0.098
LVESd (mm)	30.0 (25.0–33.0)	27.0 (23.0–31.5)	0.917
IVSd (mm)	11.0 (10.0–12.5)	9.0 (8.0–10.0)	<0.0001
LVEF (%)	60.0 (55.0–60.0)	60.0 (60.0–63.0)	0.005
MR ^a^ moderate *n* (%)	20 (32.3)	9 (9.8)	0.002
Residual interatrial shunt ^a^ *n* (%)	13 (21)	17 (18.5)	0.702
PASP ^b^ (mmHg)	40.0 (39.0–47.0)	44.0 (36.0–47.0)	0.991
PASP ≥ 40 mmHg ^b^ *n* (%)	34 (50.7)	52 (62.7)	0.143
PAcT ^b^ (ms)	104.0 (96.0–133.0)	107.0 (89.0–120.0)	0.114
TR ^b^ moderate or severe *n* (%)	34 (50.7)	25 (30.1)	0.01
Intra-atrial thrombus *n* (%)	0 (0)	0 (0)	

Data represent the number of patients (*n*), including the percentage of total number (%) or median values with corresponding interquartile range (in parenthesis). Abbreviations: IVSd—intraventricular septum wall thickness; LA—left atrial diameter; LVEDd—left ventricular end-diastolic diameter; LVEF—left ventricular ejection fraction; LVESd—left ventricular end-systolic diameter; MR—mitral regurgitation; PAcT—pulmonary artery acceleration time; PASP—pulmonary artery systolic pressure; RV—right ventricle; RVEDd—right ventricular end-diastolic diameter; TR—tricuspid regurgitation. ^a^ Data were available for 62 patients from HTN+ group and 92 patients from HTN− group. ^b^ Data were available for 67 patients from HTN+ group and 83 patients from HTN− group.

**Table 5:** Changes in echocardiographic parameters 6 months after transcatheter atrial septal defect closure (ASDC) compared to baseline values in the group of patients with systemic essential hypertension (HTN+).

Variable	Before ASDC (*n* = 79)	6 Months after ASDC (*n* = 78)	*p*-Value
RVEDd (mm)	33.0 (30.0–39.0)	30.0 (27.5–35.0) ^a^	<0.001
LA (mm)	41.5 (37.0–45.0)	42.0 (39.0–45.0)	0.352
LVEDd (mm)	44.0 (41.0–48.0)	47.0 (43.0–50.0)	0.008
LVESd (mm)	30.0 (25.0–33.0)	30.0 (27.0–33.0)	0.330
LVEF (%)	60.0 (55.0–60.0)	60.0 (60.0–60.0)	0.296
LVDD *n* (%)	28 (47.5) ^b^	18 (28.1) ^a^	0.027
MR moderate *n* (%)	24 (35.8) ^c^	16 (25) ^a^	0.179
MR moderate-to-severe or severe *n* (%)	0 (0) ^c^	3 (4.7) ^a^	0.227
PASP (mmHg)	46.0 (40.5–52.0) ^c^	40.5 (39.0–50.5) ^c^	0.025
PASP ≥ 40 mmHg *n* (%)	52 (77.6) ^c^	34 (50.7) ^c^	0.012
PAcT (ms)	96.0 (74.0–110.0) ^c^	104.0 (81.0–118.0) ^c^	0.266
TR moderate or severe *n* (%)	54 (80.6) ^c^	34 (50.7) ^c^	<0.001
Intra-atrial thrombus *n* (%)	0 (0)	2 (2.6)	0.471

Data represent the number of patients (*n*), including the percentage of total number (%) or median values with corresponding interquartile range (in parenthesis). Abbreviations: ASDC—atrial septal defect closure; LA—left atrial diameter; LVDD—left ventricular diastolic dysfunction; LVEDd—left ventricular end-diastolic diameter; LVEF—left ventricular ejection fraction; LVESd—left ventricular end-systolic diameter; MR—mitral regurgitation; PAcT—pulmonary artery acceleration time; PASP—pulmonary artery systolic pressure; RVEDd—right ventricular end-diastolic diameter; TR—tricuspid regurgitation. ^a^ Data were available for 64 patients. ^b^ Data were available for 59 patients. ^c^ Data were available for 67 patients.

**Table 6:** Comparison of echocardiographic characteristics of patients with secundum atrial septal defect and with (HTN+ group) or without (HTN− group) systemic essential hypertension at 6 months after transcatheter atrial septal defect closure.

Variable	HTN+ Group (*n* = 78)	HTN− Group (*n* = 104)	*p*-Value between Groups
RVEDd ^a^ (mm)	30.0 (27.5–35.0)	27.0 (25.0–30.0)	<0.0001
Relative RVEDd change compared to baseline ^a^ (mm)	−3.0 (−7.0–0.0)	−4.0 (−9.0–−1.0)	0.129
Reverse RV remodeling ^a^ *n* (%)	38 (59.4)	57 (67.9)	0.286
LA (mm)	42.0 (39.0–45.0)	35.0 (33.0–37.5)	<0.0001
LVEDd (mm)	47.0 (43.0–50.0)	45.0 (41.0–48.0)	0.117
LVESd (mm)	30.0 (27.0–33.0)	29.0 (26.0–33.0)	0.502
IVSd (mm)	11.0 (10.0–13.0)	10.0 (9.0–11.0)	<0.0001
LVEF (%)	60.0 (60.0–60.0)	60.0 (60.0–63.0)	0.009
LVDD ^a^ *n* (%)	18 (28.1)	9 (10.7)	0.007
MR ^a^ moderate *n* (%)	16 (25)	8 (9.5)	0.011
MR ^a^ moderate-to-severe or severe ^a^ *n* (%)	3 (4.7)	1 (1.2)	0.194
Residual interatrial shunt ^a^ *n* (%)	15 (23.4)	11 (13.1)	0.101
PASP ^b^ (mmHg)	40.5 (39.0–50.5)	38.0 (35.0–42.0)	0.083
PASP ≥ 40 mmHg ^b^ *n* (%)	34 (53.1)	26 (31.0)	0.016
PAcT ^b^ (ms)	104.0 (81.0–118.0)	126.0 (107.0–145.0)	0.002
TR ^b^ moderate *n* (%)	34 (50.7)	15 (18.1)	0.003
Intra-atrial thrombus *n* (%)	2 (2.6)	0 (0)	0.101
A composite of reverse RV remodeling and a lack of residual shunt ^a^ *n* (%)	28 (43.8)	48 (57.1)	0.106

Data represent the number of patients (*n*) including the percentage of total number (%) or median values with corresponding interquartile range (in parenthesis). Abbreviations: IVSd—intraventricular septum wall thickness; LA—left atrial diameter; LVDD—left ventricular diastolic dysfunction; LVEDd—left ventricular end-diastolic diameter; LVEF—left ventricular ejection fraction; LVESd—left ventricular end-systolic diameter; MR—mitral regurgitation; PAcT—pulmonary artery acceleration time; PASP—pulmonary artery systolic pressure; RV—right ventricle; RVEDd—right ventricular end-diastolic diameter; TR—tricuspid regurgitation. ^a^ Data were available for 64 patients from HTN+ group and 84 patients from HTN− group. ^b^ Data were available for 67 patients from HTN+ group and 83 patients from HTN− group.

**Table 7:** Transcatheter atrial septal defect closure-related complications in patients with secundum atrial septal defect and with (HTN+ group) or without (HTN− group) systemic essential hypertension.

ASDC-Related Complication	HTN+ Group (*n* = 79)	HTN− Group (*n* = 105)	*p*-Value between Groups
Total complications *n* (%)	10 (12.7)	10 (9.5)	0.513
Total minor/major complications *n* (%)	6/4 (7.6/5.1)	5/5 (4.8/4.8)	0.422
Early complications *n* (%)	7 (8.9)	8 (7.6)	0.761
Early major complications *n* (%)	1 (1.3)	3 (2.9)	0.464
Device embolization requiring urgent surgical intervention *n* (%)	1 (1.3)	1 (0.95)	
Cardiac arrest followed by resuscitation *n* (%)	0 (0)	1 (0.95)	
HF exacerbation requiring parenteral diuretic *n* (%)	0 (0)	1 (0.95)	
Early minor complications *n* (%)	6 (7.6)	5 (4.8)	0.422
Minor venous access site bleeding *n* (%)	4 (5.1)	3 (2.9)	
Pericardial effusion *n* (%)	2 (2.5)	2 (1.9)	
Transient arrhythmias *n* (%)	0 (0)	1 (0.95)	
Groin hematoma not requiring surgery *n* (%)	1 (1.3)	1 (0.95)	
Late major complications *n* (%)	3 (3.8)	2 (1.9)	0.441
HF exacerbation requiring hospitalization *n* (%)	0 (0)	1 (0.95)	
New onset of significant MR or deterioration of MR *n* (%)	3 (3.8)	1 (0.95)	

Data represent the number of patients (*n*), including the percentage of total number (%). Abbreviations: ASDC—transcatheter atrial septal defect closure; HF—heart failure; MR—mitral regurgitation.

## Data Availability

The data presented in this study are available on request from the corresponding author. The data are not publicly available due to privacy restrictions.
